# Discrimination between Highly Pathogenic and Low Pathogenic H5 Avian Influenza A Viruses

**DOI:** 10.3201/eid1204.051427

**Published:** 2006-04

**Authors:** Sunchai Payungporn, Salin Chutinimitkul, Arunee Chaisingh, Sudarat Damrongwantanapokin, Bandit Nuansrichay, Wasana Pinyochon, Alongkorn Amonsin, Ruben O. Donis, Apiradee Theamboonlers, Yong Poovorawan

**Affiliations:** * Chulalongkorn University, Bangkok, Thailand;; † National Institute of Animal Health, Bangkok, Thailand;; ‡ Centers for Disease Control and Prevention, Atlanta, Georgia, USA

**Keywords:** Avian influenza, H5N1, real-time PCR, melting curve, H5 subtypes

**To the Editor:** To thoroughly investigate avian influenza outbreaks, identifying highly pathogenic avian influenza (HPAI) and low pathogenic avian influenza (LPAI) is essential. Currently, determination of inserted basic amino acids within the hemagglutinin cleavage site of HPAI relies on nucleotide sequencing (*1**–**3*). Direct sequencing is relatively time-consuming and laborious and thus is not suitable for local and regional diagnostic laboratories that receive large numbers of samples that may contain HPAI or LPAI subtype H5N1. Therefore, a rapid diagnostic assay was developed to discriminate between HPAI and LPAI subtype H5 viruses by 1-step real-time reverse transcription–polymerase chain reaction (RT-PCR) with melting curve analysis.

H5 primers flanking the cleavage site were designed from conserved regions among HPAI and LPAI strains by using nucleotide sequences obtained from GenBank and our previous studies (*4**–**6*). The primers consisted of a forward primer H5F3+ (nucleotides 1001–1021: 5´-AACAGATTAGTCCTTGCGACTG-3´) and a reverse primer H5R2+ (nucleotides 1124–1103: 5´-CATCTACCATTCCCTGCCATCC-3´), which yielded products of ≈124 bp and ≈112 bp, corresponding to HPAI and LPAI, respectively. Consequently, the sizes of the amplicons and percentage guanine-cytosine content were different, allowing discrimination between HPAI and LPAI by melting curve analysis (*7*).

One-step, real-time RT-PCR with melting curve analysis was performed in an ABI 7500 system (Applied Biosystems, Foster City, CA, USA). In each reaction, 3.0 μL of RNA sample was combined with a reaction mixture containing 10 μL 2× SYBR Green PCR Master Mix (Applied Biosystems), 0.5 μL 40× MultiScribe (Applied Biosystems) and RNase inhibitor, each primer (at final concentration of 0.5 μmol/L), 1.5 mmol/L MgCl_2_, and RNase-free water in a final volume of 20 μL. The thermal profile began with incubation at 48°C for 45 min (reverse transcription), then incubation at 95°C for 10 min (predenaturation), followed by 40 cycles of amplification alternating between 94°C for 15 s (denaturation) and 68°C for 40 s (annealing/extension). The SYBR Green I fluorescent signal was obtained once per cycle at the end of the extension step. After amplification, melting curve analysis was performed by heating the sample to 95°C for 15 s, then cooling it to 70°C for 1 min, followed by a linear temperature increase to 95°C at a rate of 0.5°C/s, while continuously monitoring the fluorescent signal. Data were analyzed by the 7500 System SDS Software version 1.2 (Applied Biosystems).

To develop the assay, samples previously identified as influenza A/chicken/Nakorn-Patom/Thailand/CU-K2/04 (H5N1) served as a control for HPAI, and A/duck/Hong Kong/308/78 (H5N3) served as a control for LPAI. The H5 genes (nucleotides 914–1728) of each strain were inserted into pGEM-T Easy Vector and then transcribed in vitro by using RiboMAX Large Scale RNA Production System-T7 (Promega, Madison, WI, USA). Serial 10-fold dilutions of the standard H5 RNA were subjected to a sensitivity test (*8*). The fluorescent signal can be detected at RNA dilutions as low as 10^2^ copies/μL. To assess the specificity, viral RNA extracted from other subtypes of influenza A viruses (H1–H4 and H6–H15) was tested. The assay was specific for the H5 subtype, since no amplification was detected from other subtypes.

Three preliminary melting curve analyses showed that this assay was effective in discriminating between the melting peaks of HPAI and LPAI (Figure). The variations of melting temperature (T_m_) between runs were experimentally determined. The mean (standard deviation) of T_m_ values for HPAI and LPAI were 77.43°C (0.21°C) and 79.57°C (0.23°C), respectively. This assay provided high reliability and reproducibility, since the coefficients of variation were <0.30.

**Figure:**
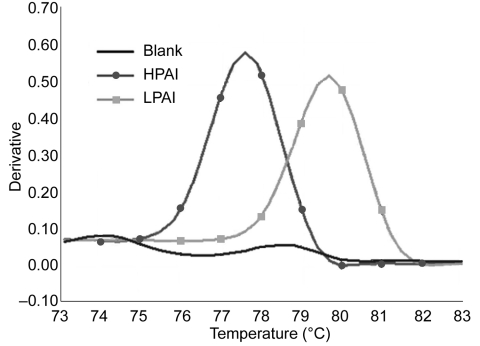
Discrimination between highly pathogenic avian influenza (HPAI) and low pathogenic avian influenza (LPAI) by melting curve analysis based on real-time reverse transcription–polymerase chain reaction of the H5 HA gene with SYBR Green I fluorescent dye. The melting peaks of HPAI and LPAI were clearly separated. The cutoff value was set at 78.50°C (midpoint between HPAI and LPAI) and used to interpret the pathogenicity of unknown samples.

Seventy-eight specimens of influenza A virus were used to validate the assay. The 75 HPAI samples were isolated during the 2004 outbreak in Thailand; 3 LPAI samples, including A/avian/NY/01 (H5N2), A/Chicken/Mexico/31381-3/94 (H5N2), and A/shoveler/Egypt/03 (H5N2), were provided by the Centers for Disease Control and Prevention. The viruses were isolated in embryonated chicken eggs as described previously (*9*). RNA was extracted from 140 μL of allantoic fluid, and RT-PCR was performed as described above. Melting curve analysis showed that all H5N1 isolates from the 2004 outbreak in Thailand were interpreted as HPAI, whereas the 3 samples of H5N2 subtype were classified as LPAI. The T_m_ varied from 77.2°C to 78.1°C for HPAI and 78.75°C to 79.5°C for LPAI. The melting curve analysis results were completely concordant with the results of direct sequence analysis of the H5 gene for all samples tested.

The melting curve analysis described here provides a rapid, accurate, and high-throughput approach to discriminate between HPAI and LPAI. This assay is particularly attractive for large-scale screening of suspected subtype H5 influenza A virus during outbreaks to identify candidate LPAI that could be used as vaccine strains.
